# Supplementary material for: Highly efficient platelet generation in lung vasculature reproduced by microfluidics
Source: Nat Commun. 2023 Jul 7;14:4026. doi: 10.1038/s41467-023-39598-9 (PMC10329040; doi:10.1038/s41467-023-39598-9)
Supplement: Supplementary file 1 — Supplementary information [file 41467_2023_39598_MOESM1_ESM.pdf]

Supplementary information

Highly efficient platelet generation in lung vasculature reproduced by microfluidics

Supplementary Method

Materials

Cell culture reagents including IMDM-Glutamax and penicillin-streptomycin were from Invitrogen (Paisley, UK). Recombinant murine stem cell factor (rSCF) and recombinant murine thrombopoietin (rTPO) were purchased from PeproTech EC (London, UK). PE or FITC-conjugated anti-CD41antibodies and isotype-nonspecific IgG were from BD Biosciences (Berkshire, UK). FITC-conjugated anti-mouse CD31, CD102 antibodies and isotype-nonspecific IgG were from Biolegend (San Diego, USA). SYLGARD™ 184 Silicone Elastomer Kit was from Dow Corning (Michigan, USA). Anti-mouse GPIbα antibody R300, FITC-conjugated anti-mouse CD61, CD42b, CD42d, CD49b, Glycoprotein VI (GPVI) antibodies and isotype-nonspecific IgG, PE-conjugated anti-CD62P antibodies, JON/A anti-integrin αIIbβ3 and isotype-nonspecific IgG were from Emfret Analytics (Würzburg, Germany). Hoechst 33342, DRAQ5, Calcein AM, Calcein Deep Red, Tetramethyl rhodamine methyl ester (TMRM) and CellTracker™ Red CMTPX dye were from Molecular Probes™ (Loughborough, UK). $DiOC_6$ was supplied by Enzo Life Sciences (Exeter, UK). Ibidi slides were from Thistle Scientific (Glasgow, UK). Horm fibrillary collagen was from Takeda Pharma (Linz, Austria). Cross-linked collagen-related peptide (CRP-XL) was provided by Richard Farndale (University of Cambridge, UK). Unless stated, all other reagents were from Sigma-Aldrich (Dorset, UK). Reagents for intravital bone marrow two-photon experiments were as follows: anti-CD105 Alexa Fluor 546 (clone MJ7/19) was purified and

labeled in house; rat monoclonal anti-mouse GPIX (CD42a) Alexa Fluor 488 was from Emfret Analytics and labeling was in house; bovine serum albumin (BSA) was from AppliChem and labeled in house.

Supplementary Figure

Supplementary Fig.1

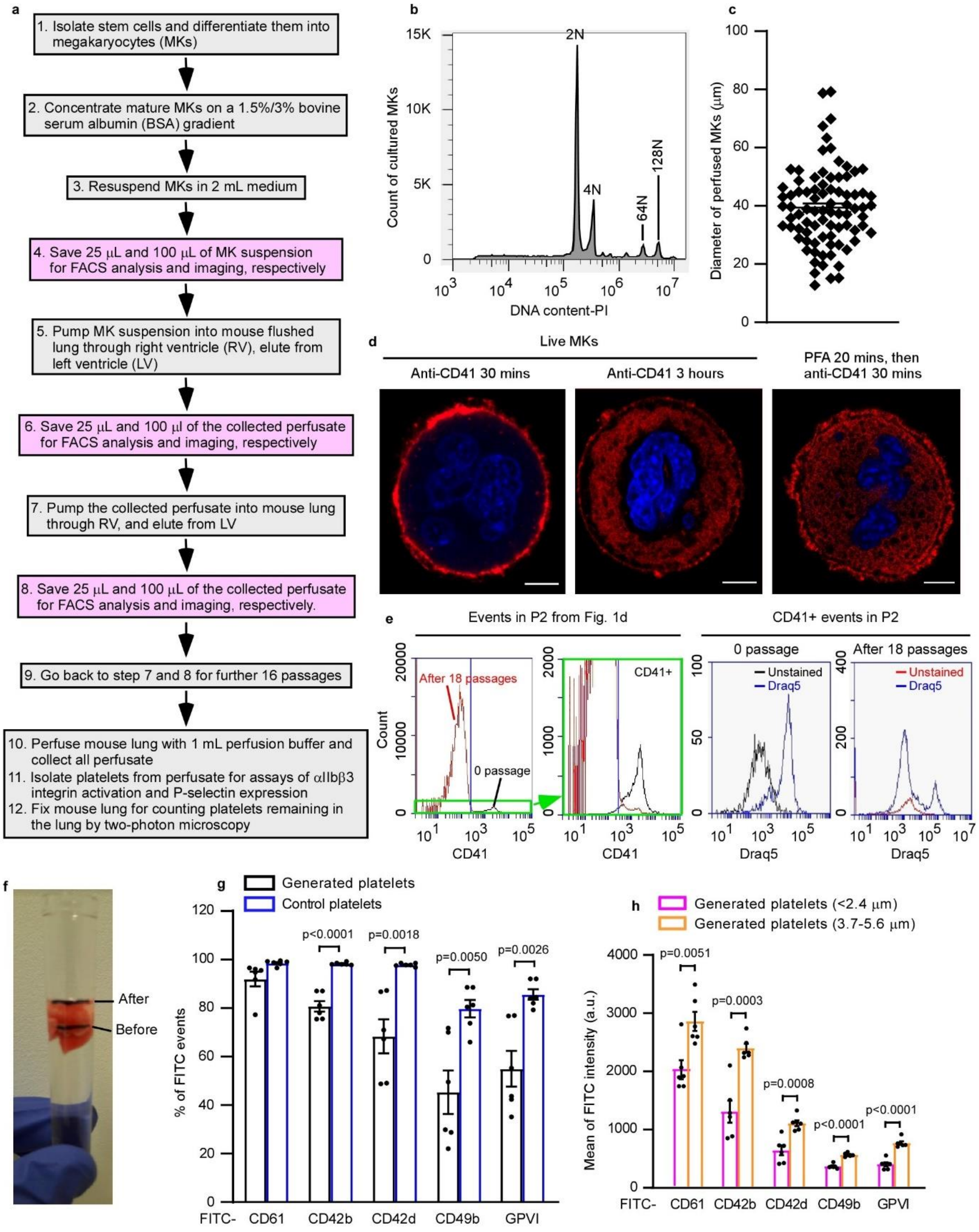

Supplementary Fig. 1: Approach to generating mouse platelets from megakaryocytes by infusion through the pulmonary vasculature. **a** Experimental flowchart for generating platelets from repeated infusion of mouse heart-lung preparation. **b** DNA ploidy analysis of cultured mouse megakaryocytes (MKs). MKs were stained with FxCycle PI/RNAse staining solution and measured by fluorescence-activated cell sorting (FACS). **c** The diameter of perfused MKs was measured using Fiji. MKs were enriched on a 1.5%/3% bovine serum albumin (BSA) gradient and stained with anti-CD41-PE and Hoechst 33342. Images were obtained on an inverted SP8 confocal microscope. Data are presented as mean ± SEM (39.4 ± 1.5 µm). The diameter of MKs (83 MKs from n=5 independent experiments) was measured using Fiji (ImageJ-Win64). **d** The demarcation membrane system (DMS) and plasma membrane of mouse MKs were stained with PE- or FITC-conjugated CD41 (red) and nuclei with Hoechst 33342 (blue). At least 50 MKs from at least 3 independent experiments (n>3) for each group were imaged. Images were obtained on an inverted SP8 confocal microscope. Incubation of live MKs with anti-CD41 stains surface receptor by 30 mins, whilst by 3 hours the DMS is also efficiently stained. DMS staining in PFA-fixed MKs is shown for comparison. Scale bar: 10 µm. **e** Example FACS histograms showed the events and CD41+ events in P2 gates from Fig. 1d at 0 passages and after 18 passages. 10 µL of MK suspension at 0 passages or perfusates after 18 passages were measured by FACS. In order to show more clearly CD41+ events in P2, the panel with green surround is a magnified histogram of events in the left-hand panel, as indicated by the green arrow. DRAQ5 staining for MKs provides a positive control for Fig. 1e and Fig. 3d. **f** The lung volume after 18 passages was estimated by a fluid displacement technique. This was used to allow accurate estimation of numbers of platelets generated that were still retained within the circulation of the lung. **g-h** Platelet surface glycoproteins were measured by FACS after staining with different FITC-conjugated antibodies

as indicated. Mouse MKs, labelled with CD41-PE antibody, were passaged through the pulmonary vasculature *ex vivo* 18 times. Lungs were ventilated with air throughout. Generated platelets were defined by staining with CD41-PE antibody. N=6 independent experiments and data are mean ± SEM. Two-tailed unpaired *t*-test. **g** % of FITC- positive events was compared between generated platelets and control platelets; $p<0.0001$ for CD42b, $p=0.0018$ for CD42d, $p=0.0050$ for CD49b and $p=0.0026$ for GPVI. **h** Surface glycoproteins of two subpopulations of generated platelets, segregated by platelet size (diameter <2.4 μm vs diameter 3.7-5.6 μm), are shown; $p=0.0051$ for CD61, $p=0.0003$ for CD42b, $p=0.0008$ for CD42d, $p<0.0001$ for CD49b and $p<0.0001$ for GPVI.
