# Supplementary material for: Highly efficient platelet generation in lung vasculature reproduced by microfluidics
Source: Nat Commun. 2023 Jul 7;14:4026. doi: 10.1038/s41467-023-39598-9 (PMC10329040; doi:10.1038/s41467-023-39598-9)
Supplement: Supplementary file 3 — Description of Additional Supplementary Files [file 41467_2023_39598_MOESM3_ESM.pdf]

Description of Additional Supplementary Files

Supplementary Movie 1. 3D reconstructed image of ex vivo mouse lung – air ventilation. The demarcation membrane system and plasma membrane of mouse megakaryocytes were stained with anti-CD41-PE (red) and nuclei stained with Hoechst 33342 (cyan) and passaged through ex vivo mouse pulmonary circulation 18 times, whilst lungs were artificially ventilated with air. Images were taken by two-photon microscopy, and reconstructions made from 20 z-stacks (in 2 µm steps) and reconstructed into 3D space shown in the video. Red events with ~2-6 µm in diameter were generated platelets. Images are representative of n=4 independent experiments. Scale bar in this video: 20 µm.

Supplementary Movie 2. 3D reconstructed image of ex vivo mouse lung – air ventilation. The demarcation membrane system and plasma membrane of mouse megakaryocytes were stained with anti-CD41-FITC (green) and passaged through ex vivo mouse pulmonary circulation 18 times, whilst lungs were artificially ventilated with air. Images were taken by two-photon microscopy, and reconstructions made from 20 z-stacks (in 2 µm steps) and reconstructed into 3D space shown in the video. Images are representative of n=6 independent experiments. Green events with ~2-6 µm in diameter were generated platelets. Scale bar in this video: 20 µm.

Supplementary Movie 3. 3D reconstructed image of ex vivo mouse control lung – air ventilation. To demonstrate background autofluorescence, lungs were artificially ventilated with air and imaged by two-photon microscopy, and reconstructions made from 20 z-stacks (in 2 µm steps) and reconstructed into 3D space shown in the video. Images are representative of n=4 independent experiments. Scale bar in this video: 20 µm.

Supplementary Movie 4. 3D reconstructed image of ex vivo mouse lung – unventilation. The demarcation membrane system and plasma membrane of mouse megakaryocytes were stained with anti-CD41-FITC (green) and passaged through ex vivo mouse pulmonary circulation 18 times, whilst lungs were not artificially ventilated. Images were taken by two-photon

microscopy, and reconstructions made from 20 z-stacks (in 2 µm steps) and reconstructed into 3D space shown in the video. Images are representative of n=4 independent experiments. Green events with ~2-6 µm in diameter were generated platelets. Scale bar in this video: 20 µm.

Supplementary Movie 5. 3D reconstructed image of ex vivo mouse lung – nitrogen ventilation. The demarcation membrane system and plasma membrane of mouse megakaryocytes were stained with anti-CD41-FITC (green) and passaged through ex vivo mouse pulmonary circulation 18 times, whilst lungs were artificially ventilated with 100% nitrogen. Images were taken by two-photon microscopy, and reconstructions made from 40 z-stacks (in 2 µm steps) and reconstructed into 3D space shown in the video. Images are representative of n=4 independent experiments. Several MKs with very bright green fluorescence are trapped in the pulmonary circulation. Scale bar in this video: 20 µm.

Supplementary Movie 6. 3D reconstructed image of in vitro thrombus formation under flow. Generated platelets (stained with DiOC6 plus CellTracker™ Red CMTPX, blue) and control platelets (stained with CellTracker™Red CMTPX alone, magenta) were mixed and perfused through the ibidi slide pre-coated with collagen, at a shear rate of 1000/s for 20 minutes. Images were taken by confocal fluorescence microscopy, and reconstructions made from 30 z-stacks (in 0.787 µm steps) and reconstructed into 3D space shown in the video. Images are representative of n=5 independent experiments. Scale bar in this video: 60 µm.

Supplementary Movie 7. 3D reconstructed confocal image of an intact megakaryocyte with a central giant lobulated nucleus surrounded by cytoplasm. The demarcation membrane system and plasma membrane of a mouse megakaryocyte stained with anti-CD41-PE (red) and nucleus stained with Hoechst 33342 (blue). Images were obtained on an inverted SP8 confocal microscope and 3D reconstruction image was made from 50 z-stacks at 2 µm z-step spacing. Images are representative of n=8 independent experiments. Scale bar in the video: 10 µm.

Supplementary Movie 8. 3D reconstructed confocal image of a megakaryocyte with an extruding giant lobulated nucleus adhering to a large cytoplasmic fragment. The demarcation membrane system and plasma membrane of a mouse megakaryocyte stained with anti-CD41-PE (red) and nucleus stained with Hoechst 33342 (blue). Images were obtained on an inverted SP8 confocal microscope and 3D reconstruction image was made from 16 z-stacks at 2 µm z-step spacing. Images are representative of n=8 independent experiments. Scale bar in the video: 10 µm.

Supplementary Movie 9. 3D reconstructed confocal image of a naked nucleus lobulated encased in thin/patchy membrane. The demarcation membrane system and plasma membrane of a mouse megakaryocyte stained with anti-CD41-PE (red) and nucleus stained with Hoechst 33342 (blue). Images were obtained on an inverted SP8 confocal microscope and 3D reconstruction image was made from 22 z-stacks at 2 µm z-step spacing. Images are representative of n=8 independent experiments. Scale bar in the video: 10 µm.

Supplementary Movie 10. 3D reconstructed confocal image of a naked lobulated nucleus with a completely separated large cytoplasmic fragment. The demarcation membrane system and plasma membrane of a mouse megakaryocyte stained with anti-CD41-PE (red) and nucleus stained with Hoechst 33342 (blue). Images were obtained on an inverted SP8 confocal microscope and 3D reconstruction image was made from 20 z-stacks at 2 µm z-step spacing. Images are representative of n=8 independent experiments. Scale bar in the video: 10 µm.

Supplementary Movie 11. 3D reconstructed confocal image of sub-nuclei. The demarcation membrane system and plasma membrane of mouse megakaryocytes (MKs) were stained with anti-CD41-PE (red) and nuclei stained with Hoechst 33342 (blue) and passaged through ex vivo mouse pulmonary circulation 3 times, whilst lungs were artificially ventilated with air. Images were obtained on an inverted SP8 confocal microscope and 3D reconstructed image was made from 60 z-stacks at 0.5 µm z-step spacing. Nuclear lobes were partially fragmenting from a naked MK nucleus. Images are representative of n=8 independent experiments. Scale bar in the video: 10 µm.

Supplementary Movie 12. 3D reconstructed image of ex vivo wild-type mouse lung infused with Tropomyosin4-/- (Tpm4-/-) megakaryocytes under air ventilation. The demarcation membrane system and plasma membrane of Tpm4-/- megakaryocytes were stained with antiCD41-FITC (green) and passaged through ex vivo wild-type mouse pulmonary circulation 18 times, whilst lungs were artificially ventilated with air. Images were taken by two-photon microscopy, and reconstructions made from 20 z-stacks at 2 µm step spacing and reconstructed into 3D space shown in the video. Abundant fluorescent objects sized ~10 µm, were visible in focus stacking of 20 continuous two-photon planes. Images are representative of n=3 independent experiments. Scale bar in this video: 20 µm.

Supplementary Movie 13. Dynamic morphologies of Tropomyosin4-/- (Tpm4-/-) megakaryocytes in calvarium bone marrow of living mice. Intravital two-photon microscopy of calvarium bone marrow where MKs and their derivatives (including platelets) were stained intravenously with anti-GPIX-AlexaFluor 488 antibody (cyan) whilst endothelial cell membranes were stained with anti-CD105-AlexaFluor 546 antibody (red). Among examples, white arrows point to large fragments of MKs within sinusoidal vessels releasing heterogeneous structures in the direction of blood flow. Yellow arrowheads show some MKs within the marrow space producing extensions into sinusoids. One MK is shown wholly within the sinusoid showing cellular extensions (indicated in circles). Images were taken from n=6 Tpm4-/- mice. Scale bars in this video: 50 µm.

Supplementary Movie 14. Dynamic morphologies of wild-type megakaryocytes in calvarium bone marrow of living mice. Intravital two-photon microscopy of calvarium bone marrow where MKs and their derivatives (including platelets) were stained intravenously with antiGPIX-AlexaFluor 488 antibody (cyan) whilst endothelial cell membranes were stained with antiCD105-AlexaFluor 546 antibody (red). Among examples, white arrows point to large fragments of MKs within sinusoidal vessels releasing heterogeneous structures in the direction of blood flow. Yellow arrowheads show some MKs within the marrow space producing extensions into

sinusoids. One MK is shown wholly within the sinusoid showing cellular extensions (indicated in circles). Images were taken from n=6 wild-type mice. Scale bars in this video: 50 µm.
