# Supplementary material for: Highly efficient platelet generation in lung vasculature reproduced by microfluidics
Source: Nat Commun. 2023 Jul 7;14:4026. doi: 10.1038/s41467-023-39598-9 (PMC10329040; doi:10.1038/s41467-023-39598-9)
Supplement: Supplementary file 18 — Reporting Summary [file 41467_2023_39598_MOESM18_ESM.pdf]

nature portfolio

Corresponding author(s): Xiaojuan Zhao & Alastair Poole

Last updated by author(s): Jun 12, 2023

Reporting Summary

Nature Portfolio wishes to improve the reproducibility of the work that we publish. This form provides structure for consistency and transparency in reporting. For further information on Nature Portfolio policies, see our Editorial Policies and the Editorial Policy Checklist.

Statistics

For all statistical analyses, confirm that the following items are present in the figure legend, table legend, main text, or Methods section.

n/a	Confirmed	
☐	☒	The exact sample size (*n*) for each experimental group/condition, given as a discrete number and unit of measurement
☐	☒	A statement on whether measurements were taken from distinct samples or whether the same sample was measured repeatedly
☐	☒	The statistical test(s) used AND whether they are one- or two-sided<br>*Only common tests should be described solely by name; describe more complex techniques in the Methods section.*
☒	☐	A description of all covariates tested
☐	☒	A description of any assumptions or corrections, such as tests of normality and adjustment for multiple comparisons
☐	☒	A full description of the statistical parameters including central tendency (e.g. means) or other basic estimates (e.g. regression coefficient) AND variation (e.g. standard deviation) or associated estimates of uncertainty (e.g. confidence intervals)
☐	☒	For null hypothesis testing, the test statistic (e.g. *F*, *t*, *r*) with confidence intervals, effect sizes, degrees of freedom and *P* value noted<br>*Give P values as exact values whenever suitable.*
☒	☐	For Bayesian analysis, information on the choice of priors and Markov chain Monte Carlo settings
☒	☐	For hierarchical and complex designs, identification of the appropriate level for tests and full reporting of outcomes
☒	☐	Estimates of effect sizes (e.g. Cohen's *d*, Pearson's *r*), indicating how they were calculated

Our web collection on statistics for biologists contains articles on many of the points above.

Software and code

Policy information about availability of computer code

Data collection	Confocal microscopy: LAS X (version 3.5.7.23225, Leica Microsystems)<br>Two-photon microscopy: LAS X (version 3.1.5.16308, Leica Microsystems)<br>Flow cytometry: BD Accuri C6 Plus Software version 1.0.23.1 (Becton Dickinson)
Data analysis	Immunofluorescence and electron microscopy image preparation and analysis: ImageJ-win 64 software<br>Flow cytometry: BD Accuri C6 Plus Software version 1.0.23.1 (Becton Dickinson)<br>Statistical analysis and graph preparation: GraphPad Prism 9.

For manuscripts utilizing custom algorithms or software that are central to the research but not yet described in published literature, software must be made available to editors and reviewers. We strongly encourage code deposition in a community repository (e.g. GitHub). See the Nature Portfolio guidelines for submitting code & software for further information.

Data

Policy information about availability of data

All manuscripts must include a data availability statement. This statement should provide the following information, where applicable:

- Accession codes, unique identifiers, or web links for publicly available datasets
- A description of any restrictions on data availability
- For clinical datasets or third party data, please ensure that the statement adheres to our policy

The datasets generated during and/or analysed during the current study are included as Source Data. Source data are provided with this paper.

Research involving human participants, their data, or biological material

Policy information about studies with human participants or human data. See also policy information about sex, gender (identity/presentation), and sexual orientation and race, ethnicity and racism.

Reporting on sex and gender	Not applicable
Reporting on race, ethnicity, or other socially relevant groupings	Not applicable
Population characteristics	Not applicable
Recruitment	Not applicable
Ethics oversight	Not applicable

Note that full information on the approval of the study protocol must also be provided in the manuscript.

Field-specific reporting

Please select the one below that is the best fit for your research. If you are not sure, read the appropriate sections before making your selection.

☒ Life sciences ☐ Behavioural & social sciences ☐ Ecological, evolutionary & environmental sciences

For a reference copy of the document with all sections, see nature.com/documents/nr-reporting-summary-flat.pdf

Life sciences study design

All studies must disclose on these points even when the disclosure is negative.

Sample size	All of our datasets are from at least 3 independent experiments, and more usually 4 or greater. The final n number is determined by knowledge of the literature using similar assays, in order to derive data that achieve statistical significance for the effects sizes likely to be seen in our assays.
Data exclusions	No data were excluded from the analysis.
Replication	Each experiment was conducted at least 3 times, and usually 4 or more times, with similar results. All experiments were able to be replicated.
Randomization	Randomisation was not used because cells analysed in each group were from genetically identical sources.
Blinding	Blinding was not used in this study.

Reporting for specific materials, systems and methods

We require information from authors about some types of materials, experimental systems and methods used in many studies. Here, indicate whether each material, system or method listed is relevant to your study. If you are not sure if a list item applies to your research, read the appropriate section before selecting a response.

Materials & experimental systems

n/a	Involved in the study
☐	☒ Antibodies
☒	☐ Eukaryotic cell lines
☒	☐ Palaeontology and archaeology
☐	☒ Animals and other organisms
☒	☐ Clinical data
☒	☐ Dual use research of concern
☒	☐ Plants

Methods

n/a	Involved in the study
☒	☐ ChIP-seq
☐	☒ Flow cytometry
☒	☐ MRI-based neuroimaging

Antibodies

Antibodies used

1. PE- conjugated anti mouse-CD41 from BD Biosciences (Berkshire, UK), clone: MWReg30, Cat no.: 558040, Lot no.: 1025893 113. 1:100 imaging and FACS
2. FITC -conjugated anti mouse-CD41from BD Biosciences (Berkshire, UK). clone: MWReg30, Cat no.: 553848, Lot no.: 7306661. 1:100 imaging and FACS

3. FITC-conjugated anti-mouse CD31 from Biolegend (San Diego, USA). Clone:390. Cat no.: 102406, Lot no.: B246758. 1:100 FACS

4. FITC-conjugated anti-mouse CD102 from Biolegend (San Diego, USA). Clone:390. Cat no: 3C4 (MIC2/4), Lot no: B244384. 1:100 FACS

5. Anti-mouse GPIb antibody from Emfret Analytics (Würzburg, Germany). Clone: polyclones, Cat no: R300, Lot no: 300-Q. 2 μg/g bodyweight, intraperitoneal administration

6. FITC-conjugated anti-mouse CD61from Emfret Analytics (Würzburg, Germany). Clone: Luc. H11, Cat no: M031-1, Lot no: 0311-C. 1:100 FACS

7. FITC-conjugated anti-mouse CD42b from Emfret Analytics (Würzburg, Germany). Clone: Xia.G5, Cat no: M040-1, Lot no: 0401-D. 1:100 FACS

8. FITC-conjugated anti-mouse CD42d from Emfret Analytics (Würzburg, Germany). Clone: Gon.C2, Cat no: M060-1, Lot no: 0601-B. 1:100 FACS

9. FITC-conjugated anti-mouse CD49b from Emfret Analytics (Würzburg, Germany). Clone: Sam.C1, Cat no: M071-1, Lot no: 0711-A. 1:100 FACS

10. FITC-conjugated anti-mouse Glycoprotein VI from Emfret Analytics (Würzburg, Germany). Clone: JAQ1, Cat no: M040-1, Lot no: 03401-D. 1:100 FACS

11. PE-conjugated anti mouse-CD62P antibodies from Emfret Analytics (Würzburg, Germany). Clone: Wug. E9, Cat no.: M130-2, Lot no: M1302-D. 1:100 FACS

12. PE-conjugated anti mouse integrin αIIbβ3 activation from Emfret Analytics (Würzburg, Germany). Clone: JON/A, Cat no.: M023-2, Lot no: M0232-G. 1:100 FACS

13. Anti mouse CD105 Alexa Fluor 546 (clone: MJ7/19) was purified and labeled in house. 1 μg/g body weight, intravenous injection

14. Anti-mouse GPIX (CD42a) Alexa Fluor 488 (clone: Xia.B4, Cat no.: M051-0) was from Emfret Analytics and labeling was in house. 1.5 μg/g body weight, intravenous injection

Validation

PE- or FITC- conjugated anti mouse-CD41 for FACS and imaging.
FITC-conjugated anti-mouse CD31, CD102, CD61, CD42b, CD42d, CD49b, anti-mouse Glycoprotein VI for FACS. Anti-mouse GPIb antibody R300 for mouse platelet depletion. PE-conjugated anti mouse-CD62P and anti mouse-integrin αIIbβ3 antibodies for FACS. Validation was provided through the manufacturer's data sheet (see above for manufacturer and product code information) and/or the published use and validation of the relevant antibodies by our and other research groups.

Animals and other research organisms

Policy information about studies involving animals; ARRIVE guidelines recommended for reporting animal research, and Sex and Gender in Research

Laboratory animals

Mice, C57BL/6, M or F, 8-12 weeks of age, including wild-type and Tpm4-/- mice.
The mice were maintained in cages with ad libitum access to water and standard food, which were located in a 12 h light/dark cycle animal room with room temperature ~22 °C, and humidity of 40-60%.

Wild animals

Not applicable.

Reporting on sex	Both female and male mice were used at an age of 8-12 weeks (no selection for sex of mice).
Field-collected samples	Not applicable.
Ethics oversight	The study protocol of C57BL/6 mouse care and experiments was approved by the University of Bristol local research ethics committee (AWERB) and licensed under UK Home Office project license PPL 30/3445 and PP5643338. The study protocol of Tpm4-/- mice was approved by the district government of lower Franconia, Germany (Bezirksregierung Unterfranken).

Note that full information on the approval of the study protocol must also be provided in the manuscript.

Flow Cytometry

Plots

Confirm that:

☒ The axis labels state the marker and fluorochrome used (e.g. CD4-FITC).

☒ The axis scales are clearly visible. Include numbers along axes only for bottom left plot of group (a 'group' is an analysis of identical markers).

☐ All plots are contour plots with outliers or pseudocolor plots.

☐ A numerical value for number of cells or percentage (with statistics) is provided.

Methodology

Sample preparation	Platelets derived from IgG-PE or IgG-FITC stained MKs were set up as negative controls. 25 µL of CD41-PE or CD41-FITC stained MKs suspension, or perfusates from lung or suspensions from microfluidic chambers after 1, 2, 3, 6, 9, 12, 15 or 18 passages, were collected and measured by FACS. DNA content, viability and mitochondrial membrane potential of generated platelets were determined by Draq5, Calcein AM and TMRM staining, respectively.<br>To detect the viability and mitochondrial membrane potential of pulmonary endothelial cells (ECs), assays for Calcein Deep Red retention in ECs and TMRM accumulation in active mitochondria were conducted. Pulmonary ECs were isolated from perfused lungs under air- or pure-N2-ventilation or without ventilation for approximately 2 hours and marked with either anti-CD31/PECAM-1 or anti-CD102/ICAM-2 antibodies, followed by loading with Calcein Deep Red and TMRM dyes, respectively, for 25 mins at room temperature. Pulmonary ECs from fresh lungs served as control.<br>Platelet surface glycoproteins were measured by incubating with FITC-conjugated anti-mouse CD61, CD42b, CD42d, CD49b, Glycoprotein VI (GPVI) antibodies and isotype-nonspecific IgG for 20 mins at room temperature before fixation.<br>To investigate the function of generated platelets (generated platelets derived from CD41-FITC stained MKs), assays for αIIbβ3 integrin activation (JON/A-binding) and P-selectin exposure were performed.<br>Washed platelets were stimulated with 2 U/mlLthrombin or 5 µg/mL CRP for 10 mins followed by incubation with PE-JON/A or PE-P selectin antibodies for 20 mins at room temperature before fixation. Tirofiban and PE-IgG were used to exclude nonspecific binding for the measurement of PE-conjugated JON/A or PE-conjugated P-selectin exposure, respectively. Resting platelets served as negative controls.
Instrument	Samples were analysed on a BD Accuri™ C6 Plus flow cytometer (BD).
Software	BD Accuri C6 Plus Software version 1.0.23.1 (Becton Dickinson)
Cell population abundance	Platelets and megakaryocytes were defined by expression of CD41. Viability was determined by retention of Calcein AM. Mitochondrial membrane potential was assessed using TMRM. Endothelial cells were identified by anti-CD31 and CD102.
Gating strategy	Whole mouse blood and washed mouse naive platelets were used to set gates for generated platelets (P1) at the FSC/SSC density plot by size and granularity. Tirofiban and PE-IgG were used to exclude nonspecific binding for the measurement of PE-conjugated JON/A or PE-conjugated P-selectin exposure, respectively. Resting platelets served as negative controls.

☐ Tick this box to confirm that a figure exemplifying the gating strategy is provided in the Supplementary Information.
